# Medigap-guaranteed issue associated with Medicare Advantage disenrollment for beneficiaries administered a part B drug

**DOI:** 10.1093/haschl/qxae136

**Published:** 2024-10-23

**Authors:** Angela Liu, David Pittman, Gerard Anderson, Jianhui Xu

**Affiliations:** Department of Health Policy and Management, Johns Hopkins Bloomberg School of Public Health, Baltimore, MD 21205, United States; Department of Health Policy and Management, Johns Hopkins Bloomberg School of Public Health, Baltimore, MD 21205, United States; Department of Health Policy and Management, Johns Hopkins Bloomberg School of Public Health, Baltimore, MD 21205, United States; Department of Health Policy and Management, Johns Hopkins Bloomberg School of Public Health, Baltimore, MD 21205, United States

**Keywords:** Medicare Advantage, Medigap, Medicare switching, part B physician-administered drugs

## Abstract

While many Medicare beneficiaries are enrolling in Medicare Advantage (MA), some beneficiaries may want to return to traditional Medicare and purchase Medigap, especially beneficiaries who have greater medical needs. Beyond minimal federal regulations, states impose additional regulations that impact Medigap affordability. Beneficiaries in some states have greater difficulty obtaining Medigap coverage because the states where they live allow Medigap insurers to experience rate the beneficiary, which can make Medigap insurance prohibitively expensive. We examined beneficiaries who received physician-administered drugs, which can be expensive and subject to high cost sharing, to see if disenrollment from MA for these beneficiaries was greater in states with Medigap consumer protection policy levels. In 2020, we find a 1.0% average baseline average probability of MA disenrollment. For beneficiaries who received a physician-administered drug in our sample, the probability of MA disenrollment is 3.7 (95% CI, 2.6-4.8; *P* < .001) percentage points higher in Medigap-guaranteed issue states compared with states with no protections. We find a greater association between MA disenrollment and Medigap protection policies with higher cost drugs. These findings suggest that beneficiaries who receive a high-volume and high-spending physician-administered drug are more likely to disenroll from MA back to traditional Medicare when Medigap is more affordable.

## Introduction

The “Medicare Advantage Trap” describes a situation where Medicare Advantage (MA) beneficiaries may want to disenroll from MA into traditional Medicare but face policies at the state level that ultimately contribute to their decision to remain in MA.^1-3^ In traditional Medicare, beneficiaries have the option of purchasing Medicare supplemental insurance coverage (Medigap), which is additional insurance offered by a private insurer to help cover out-of-pocket costs in traditional Medicare.^4^ When a beneficiary first ages into Medicare during a 1-time initial 6-month enrollment period, Medigap is offered at a community rate.^5^ That is, the premium to purchase Medigap is a uniform rate based on the health status of the community, meaning beneficiaries with lower expected medical needs pay the same uniform rate as beneficiaries with higher expected medical needs, in an effort to be balanced.^6^ However, outside of the initial enrollment period or once a beneficiary decides to enroll in MA, then state policies determine the rules that govern their ability to purchase Medigap coverage.^7^

Except in 8 states or under special circumstances, Medigap policies are no longer community rated after the initial enrollment period. Instead, beneficiaries are offered a Medigap premium based on the beneficiary's health status, and beneficiaries could be denied coverage. This is known as experience rating, which uses medical underwriting to determine a beneficiary's premium. Beneficiaries enrolled in MA with high healthcare needs or who are otherwise unhappy with their MA coverage may wish to enroll in traditional Medicare and purchase Medigap. The use of Medigap experience rating can make the Medigap premium prohibitively expensive.^8^ These high cost/high need beneficiaries, even if they prefer to enroll in traditional Medicare and purchase Medigap, are “trapped” in MA coverage, partially due to Medigap state insurance policies.^1-3^

The actuarial reasons for the Medigap insurers not wanting to enroll beneficiaries with high medical needs are clear. These beneficiaries are most likely to incur high medical spending, and Medigap insurers are required to pay using Medicare rules and cannot utilize many of the actions that MA plans can use to control spending, such as step therapy or prior authorization for drugs. While the use of experience rating allows Medigap insurers to charge higher premiums for beneficiaries with high expected costs, it prevents these same beneficiaries from returning to traditional Medicare.

Four states have enacted policies that require Medigap insurers to sell Medigap coverage at a community rate but fall short of requiring insurers to sell Medigap (herein referred to as community rate); 4 additional states require Medigap insurers to sell Medigap at a community rate *and* require insurers to provide the product (guaranteed issue and community rate, herein referred to as guaranteed issue).^7^ During the time period of our study, state-level changes to Medigap policies were not observed.

A previous paper did not find a statistically significant difference in MA disenrollment rate by state-level Medigap policies. Instead, the authors found that beneficiaries who disenrolled from MA into traditional Medicare enrolled back into MA the next year at a higher rate for states with no Medigap consumer protection policies.^9^ This suggests that beneficiaries are seeking Medigap coverage but are not aware of the Medigap premium pricing policies. However, this paper did not examine beneficiary utilization of healthcare services prior to disenrollment.

Not all beneficiaries wanting to return to traditional Medicare are likely to experience high premiums under experience rating. Previous research finds that high-risk beneficiaries are more likely to switch out of MA,^10,11^ signaling that high-risk beneficiaries are seeking different insurance coverage. These beneficiaries are also subject to low Medigap affordability, since only those with high-risk profiles are likely to experience elevated Medigap premiums. This paper builds on the existing literature by leveraging the MA encounter data to examine disenrollment rates from MA to traditional Medicare coverage for beneficiaries who receive a high-spending part B physician-administered drug (herein referred to as a physician-administered drug).

In this analysis, the indicator of a beneficiary with high spending profile is a beneficiary receiving a top 10 highest-spending per beneficiary physician-administered drug. Total physician-administered drug spending in 2021 on traditional Medicare beneficiaries was $33 billion, and the physician-administered drug spending per enrollee grew at 9.2% annually.^12^ Roughly 1 in 10 beneficiaries who received a physician-administered drug (0.4 million beneficiaries) had at least $5000 in cost-sharing liability.^13^ However, some drugs are more expensive than the average. For example, the average 20% cost-sharing liability in traditional Medicare for the commonly prescribed cancer drug, Opdivo, is $10 200 annually. The average 20% cost-sharing liability in traditional Medicare for Avastin is $900 annually.^13^

Out-of-pocket spending can be greater for beneficiaries enrolled in MA than traditional Medicare if the beneficiary has Medigap coverage. During the time of this study (2019), beneficiaries in MA were generally responsible for 20% co-insurance for physician-administered drugs, up to an annual out-of-pocket maximum of $6700 (in-network care) and $10 000 (out-of-network care), and a small percentage of MA plans provide more generous cost sharing.^14^ In 2023, the out-of-pocket limits increased to $8850 and $13 300 for in-network and out-of-network care, respectively.^15^ Traditional Medicare beneficiaries face the same cost-sharing liability of 20% co-insurance with no out-of-pocket cap. The difference is that traditional Medicare beneficiaries can purchase a Medigap policy that covers the 20% coinsurance for part B services, which would provide substantial financial assistance for some physician-administered drugs.

To provide an example of a beneficiary's Medicare coverage choices, assume the beneficiary is taking Opdivo and has a 20% cost-sharing liability in traditional Medicare. The average annual out-of-pocket cost is $10 200.^13^ Assuming the cost of the drug is the same in MA, a beneficiary enrolled in MA during the time of our study would hit and be responsible for the out-of-pocket maximum of $6700 (assuming in-network care). On the other hand, a beneficiary enrolled in traditional Medicare with Medigap plan F would have their part B coinsurance, part B deductible, and part B excess charge 100% covered. Thus, the beneficiary's out-of-pocket expenses would be the Medigap premium. This premium varies by private insurance company. Assuming a premium of $200/month, the out-of-pocket costs for a beneficiary with traditional Medicare and Medigap plan F would be $2400 annually. Regardless of the exact Medigap premium, 1 advantage of the Medigap option is the stability to forecast healthcare costs and spending. For many beneficiaries, this is highly advantageous.

We identify MA beneficiaries who incurred an administration of one of the top 10 highest-spending per beneficiary physician-administered drugs in 2019 and examine their MA disenrollment into traditional Medicare in the following year (2020). We compare the rates of disenrollment between states with no Medigap consumer protection policies and states with Medigap consumer protection policies, stratified by beneficiaries who did and did not receive a high-spending physician-administered drug in our sample. Since many of these drugs are chronic and result in high out-of-pocket costs to the beneficiary, the hypothesis is that there will be a higher rate of disenrollment in states with Medigap consumer protection policies compared with states without Medigap consumer protection policies. We hypothesize further that this rate would differ when stratified by beneficiaries who receive a physician-administered drug. We then stratify our analysis by drug, to assess the relationship between MA disenrollment and Medigap consumer protection policies for drugs with different out-of-pocket spending levels.

## Data and methods

### Data and sample

This retrospective, encounter-based analysis combined the 2019-2020 Medicare Beneficiary Summary File and the 2019 20% nationally representative MA carrier and outpatient encounter data. The 2019 Medicare Beneficiary Summary File was used to identify beneficiaries ages 65 and older; residents of the 50 US states or Washington D.C.; alive for the duration of 2019; not dual eligible; and enrolled in an MA plan for the entire year. These beneficiaries were followed through 2020, and their Medicare coverage type was identified using the 2020 Medicare Beneficiary Summary File to determine if they stayed in MA during the year or disenrolled from MA to traditional Medicare (Appendix Figure S1).

The 2019 20% MA outpatient and carrier encounter data were used to identify beneficiaries who received an administration of one of the top 10 highest-spending per beneficiary physician-administered drugs in traditional Medicare. These drugs were identified through the publicly available Medicare Part B spending dashboard,^13,16^ and the drugs were included in our sample if they met a minimum volume threshold of 16 500 beneficiaries (see Appendix Methods S1 for minimum volume calculation). The following drugs were included: Opdivo, Keytruda, Herceptin, Orencia, Alimta, Rituxan, Xolair, Gammaked, Privigen, and Infliximab. Appendix Table S1 provides information for each drug, including the associated Healthcare Common Procedure Codes used to identify the drug.

Using publicly available data on state-level policies,^7^ all states were grouped into 1 of 3 categories: no Medigap consumer protection policies (42 states plus the District of Columbia), community rating (4 states—Washington, Minnesota, Vermont, and Arkansas), or guaranteed issue (4 states—Maine, New York, Massachusetts, and Connecticut) (see Appendix Figure S2 for the states in each category).

### Key variables

The main dependent variable was a binary indicator describing whether the beneficiary was enrolled in MA for all 12 months in 2019 but not in 2020 (“MA disenrollment”). The main independent variable was the state-level Medigap protection policy in the state in which the beneficiary resided. This is a categorical, ordinal variable, where the states with no Medigap consumer protection policies served as the reference group. In our analysis, we stratified our sample based on whether the beneficiary was administered one of the high-spending drugs in our sample in 2019. We compared MA disenrollment by state Medigap policy and receipt of administered drug in our sample.

### Covariates

The analysis controlled for beneficiary sex (male or female), race (as defined by the Research Triangle Institute algorithm) grouped into non-Hispanic White, Black, Hispanic, and Other, age (categorized into 5 groups: 65-70, 71-75, 76-80, 81-85, and 86+), comorbidity as measured by the Elixhauser Comorbidity Index,^17^ and zip code-level variables including the percentage of individuals 65 and over that were below 100% of the federal poverty line and the percentage that graduated high school. Zip code-level covariates were drawn from the 2016-2020 American Community Survey.

### Statistical analysis

First, we examined the raw MA disenrollment rates stratified by the physician-administered drug utilization and Medigap consumer protection policy state type. Second, unadjusted and adjusted linear probability model regressions were used to examine the association of state-level Medigap protections on MA disenrollment. The regressions were stratified by beneficiaries who did and did not receive an administration of one of the 10 physician-administered drugs in our sample. The Appendix Methods S1 section provides the functional form of our models and additional descriptions. Third, adjusted linear probability models were stratified by the specific drug administered, to test the association of MA disenrollment and state-level Medigap protections for drugs with different level of out-of-pocket responsibility. The hypothesis was that drugs with higher spending per beneficiary (and thus, higher out-of-pocket costs) would generate a greater association of MA disenrollment in states with Medigap-guaranteed issue compared with states with no Medigap consumer protection policies.

### Limitations

This study has limitations. First, MA beneficiaries may be considering multiple factors aside from drug coverage when deciding to disenroll from MA, eg, greater access to providers. These factors are outside the scope of this study. Given these potential other factors, this paper does not draw causal inference between the receipt of a physician-administered drug and MA disenrollment. Second, this paper compares MA disenrollment in Medigap consumer protection states to no protection states by beneficiaries who do and do not receive an administered drug in our sample. Our sample includes the top 10 highest-spending per beneficiary drugs that meet a minimum volume threshold. Beneficiaries who may have received a drug that did not meet our volume threshold would be included in the group who “did not receive an administered drug in our sample.” Some of these drugs could have high or higher spending per beneficiary than the drugs included in our sample but did not make the minimum number threshold. This limitation biases our finding toward the null. Finally, the MA encounter data provide little ability to study patient-centered outcomes or explore qualitative work around MA disenrollment. While qualitative work is important for future work, this analysis provides empirical evidence that beneficiaries who are subject to a high-spending healthcare event disenroll at differential rates, which is important to establish.

Analyses were conducted in Stata version 16 (StataCorp LLC) and R version 4.2.2. This study was approved by the Johns Hopkins Bloomberg School of Public Health Institutional Review Board (approval #11318).

## Results

After applying the exclusion criteria, 2.8 million beneficiaries were included in our sample (Appendix Figure S1). Appendix Figure S2 illustrates a map with the geographical distribution of states and their Medigap consumer protection policies. Table 1 illustrates the number of beneficiaries by state-level group. Of the 2.8 million beneficiaries enrolled in MA at the beginning of 2020, 2.4 million lived in states with no Medigap consumer protection policies, 152 040 lived in states with community rating, and 255 167 lived in states with guaranteed issue. Appendix Table S2 illustrates the number of beneficiaries by receipt of a physician-administered drug included in our sample. Of the 2.8 million beneficiaries, 12 064 beneficiaries (0.5%) were administered one of the physician-administered drugs in our sample.

**Table 1.** Counts and percentages of Medicare Advantage beneficiaries who disenrolled from Medicare Advantage by Medigap consumer protection policy state type, 2020.

	State with no protections	Community rating state	Guaranteed issue state	Total
Disenroll, *N* (%)	23 315 (0.97%)	1618 (1.06%)	3360 (1.32%)	28 293 (1.01%)
Did not disenroll, *N* (%)	2 384 121 (99.03%)	150 422 (98.94%)	251 807 (98.68%)	2 786 350 (98.99%)
Total, *N*	2 407 436	152 040	255 167	2 814 643

**Source:** Medicare Beneficiary Summary File (2019-2020) and publicly available information on Medigap consumer protection policy state type.

At the end of 2020, 28 293 (or 1.0%) beneficiaries disenrolled from MA into traditional Medicare (Table 1). Table 2 shows the rate of MA disenrollment by state categories and whether the beneficiary was administered a physician-administered drug in our sample. In states with no protections, beneficiaries who received a physician-administered drug disenrolled at a similar rate as beneficiaries who did not receive a physician-administered drug in our sample (1.0% and 1.1%, respectively). In states with community rating only, beneficiaries who received a physician-administered drug disenrolled at a higher rate (2.8%) than beneficiaries who did not receive a physician-administered drug in our sample (1.1%). In states with guaranteed issue, 4.8% of beneficiaries disenrolled from MA for those that did receive a physician-administered drug, compared with 1.3% for those who did not receive a physician-administered drug in our sample.

**Table 2.** Within each Medigap state type and receipt of drug category, this table provides the number of beneficiaries and percentage disenrolled from Medicare Advantage, as well as descriptive statistics for beneficiary-level and zip code-level covariates.

	Guaranteed issue state		Community rating state		State with no protections	
	Top 10 part B drug taken	No top 10 part B drug taken	Top 10 Part B drug taken	No top 10 Part B drug taken	Top 10 Part B drug taken	No top 10 Part B drug taken
*N* (%)	1105 (0.04%)	254 062(9.03%)	555 (0.02%)	136 264(4.84%)	10 404 (0.37%)	2 412 253(85.76%)
Disenrolled, %	4.80%	1.30%	2.78%	1.06%	1.09%	0.97%
Age, %						
65-70	24.25%	25.37%	21.73%	26.96%	24.83%	24.84%
71-75	29.95%	30.20%	37.75%	31.45%	30.11%	30.12%
76-80	22.08%	21.15%	21.73%	21.10%	21.59%	21.60%
81-85	12.49%	12.49%	11.76%	11.48%	12.92%	12.91%
>86	11.22%	10.79%	7.03%	9.00%	10.56%	10.54%
Sex, %						
Male	41.18%	44.00%	42.81%	44.95%	40.64%	44.13%
Female	58.82%	56.00%	57.19%	55.05%	59.36%	55.87%
Race, %						
White	83.62%	78.16%	91.50%	90.43%	80.83%	75.78%
Black	7.60%	9.05%	2.29%	2.47%	8.26%	9.57%
Hispanic	3.35%	5.55%	0.82%	1.40%	6.17%	8.50%
Other	5.43%	7.23%	5.39%	5.69%	4.75%	6.16%
Elixhauser Comorbidity Index, mean (SD)	6.24 (3.31)	3.37 (2.74)	5.62 (3.06)	2.77 (2.60)	6.59 (3.47)	3.70 (2.95)
Socioeconomic factors (zip code level)						
High school graduation rate, %	87.15%	85.45%	91.31%	90.99%	86.57%	85.57%
Below federal poverty level, %	8.00%	9.15%	7.52%	7.32%	8.52%	9.03%

**Source:** The data were drawn from the 2019 Medicare Beneficiary Summary File (beneficiary-level covariates), and the 2016-2020 American Community Survey (zip code-level covariates).

Examining beneficiary characteristics revealed that beneficiaries who received a drug in our sample are younger, more likely to be female, and have a higher Elixhauser score (Table 2). This is expected given that many of these drugs treat diseases with higher prevalence in females (eg, osteoporosis) and receiving a drug is suggestive of greater health needs.

**Figure 1.**
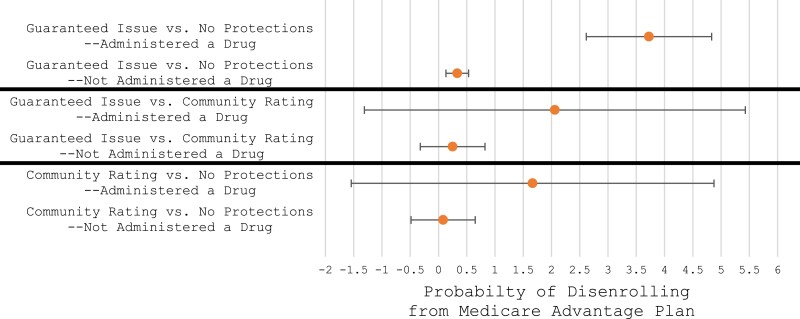
Coefficient plot of linear probability regression results stratified by beneficiaries who did and did not receive a physician-administered drug in our sample. **Source:** Medicare Advantage carrier and outpatient encounter records (2019) and Medicare Beneficiary Summary File (2019-2020).

Figure 1 illustrates a coefficient plot of the linear probability model regression results. For beneficiaries who received a physician-administered drug, the probability of MA disenrollment is 3.7 (95% CI, 2.6-4.8; *P* < 0.001) percentage points higher for beneficiaries in guaranteed issue states compared with beneficiaries in a state with no protections. The baseline average probability of MA disenrollment is 1.0%. Appendix Table S3 provides full regression results.

For beneficiaries who did not receive a physician-administered drug in our sample, the probability of MA disenrollment is 0.3 (95% CI, 0.1-0.5; *P* < 0.002) percentage points higher for beneficiaries in guaranteed issues states compared with beneficiaries in states with no Medigap protections. To interpret these findings, beneficiaries who received a physician-administered drug are 3.4 percentage points more likely than beneficiaries who did not receive a physician-administered drug in our sample to disenroll from MA in guaranteed issue states compared with states with no protections.

**Figure 2.**
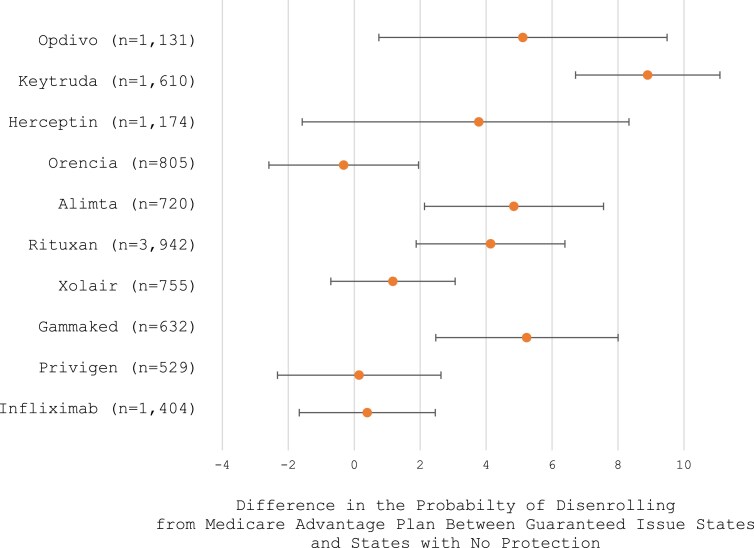
Coefficient plot of linear probability results stratified by specific physician-administered drug. **Source:** Medicare Advantage carrier and outpatient encounter records (2019) and Medicare Beneficiary Summary File (2019-2020). **Notes:** The individual drugs are listed in decreasing average annual cost-sharing liability (Appendix Table S1). These are the 10 highest-spending per beneficiary drugs.

When examining the association between Medigap state-level protections and MA disenrollment stratified by the specific physician-administered drug, we find that the association was greater with higher-cost drugs. Appendix Table S2 shows the number of beneficiaries for each of the 10 physician-administered drugs in our sample. Figure 2 illustrates the coefficients for MA disenrollment in guaranteed issue states compared with states with no Medigap protections for each of the top 10 highest-spending per beneficiary drugs, and the vertical axis orders the drugs in decreasing average annual patient out-of-pocket liability (see each drug's patient liability in Appendix Table S1).

Beneficiaries who were administered Keytruda and Opdivo had the greatest association between MA disenrollment and state-level Medigap policies. For example, for beneficiaries who were administered Keytruda, the probability of MA disenrollment was 8.9 (95% CI, 6.7-11.1; *P* < 0.001) percentage points higher for beneficiaries in guaranteed issue states compared with beneficiaries in states with no protections. For beneficiaries who were administered Opdivo, the probability of MA disenrollment was 5.1 (95% CI, 0.7-9.5; *P* = 0.023) percentage points higher for beneficiaries in guaranteed issue states compared with beneficiaries in states with no protections. Keytruda and Opdivo have high out-of-pocket cost sharing, and we found a greater association between MA disenrollment and Medigap state-level protection policies. On the other hand, drugs with relatively smaller out-of-pocket costs had a smaller association. For beneficiaries who were administered infliximab, the probability of MA disenrollment was only 0.4 (95% CI, −1.67 to 2.5; *P* = 0.701) percentage points higher for beneficiaries in guaranteed issue states compared with beneficiaries in states with no protections, and this was not statistically significant.

## Discussion

In 2020, roughly 1% of beneficiaries disenrolled from MA to traditional Medicare, which is lower than previous findings.^18,19^ This low rate of disenrollment may suggest that MA beneficiaries are satisfied with their MA plan. However, it may also suggest that MA beneficiaries are “trapped” in their plan.

When beneficiaries are stratified by those who do and do not receive a physician-administered drug in our sample, the association between MA disenrollment and state-level policy is different. Specifically, for beneficiaries who received a physician-administered drug, the probability of MA disenrollment is 3.7 percentage points higher for beneficiaries in guaranteed issue states compared with beneficiaries in a state with no protections. For beneficiaries who do not receive a physician-administered in our sample, the probability of MA disenrollment is 0.3 percentage points higher, representing a 3.4 percentage point difference. These findings are contextualized with baseline average MA disenrollment rate of 1.0%.

We find that beneficiaries are more likely to disenroll from MA in guaranteed issue states when the administered drug incurs higher spending per beneficiary (and thus, the beneficiary is subject to higher out-of-pocket cost-sharing responsibilities). Additionally, greater disenrollment was observed in Medigap-guaranteed issue states compared with community rating states, suggesting that community rating alone and the lack of guaranteed issue protections may make it difficult for beneficiaries to obtain financially accessible Medigap coverage.

It is helpful to contextualize our finding with baseline switching. We find that roughly 1% of beneficiaries switch from MA to traditional Medicare during our study period, so a 3.7 percentage point increase roughly quadruples the probability of switching. We would expect the year-over-year implications of a 3.7 percentage point increase probability in switching would be larger, since our study examines only 1 year of switching. Moreover, the population residing in restrictive Medigap states who would want to switch into traditional Medicare is likely to be larger, since some people may be deterred. Beneficiaries who remain in MA with high health needs may face additional challenges with accessing health professionals, given MA's networks and prior authorization requirements.

These empirical findings suggest that Medigap consumer protection policies are associated with MA disenrollment for beneficiaries who receive a high-spending physician-administered drug. Future work should examine other “health shocks” beyond a physician-administered drug, such as costly emergency department visits or lengthy inpatient stays. Policies to address “MA trap” should balance (1) improving beneficiary access to Medicare coverage by regulating guaranteed issue and community rating, while (2) monitoring adverse selection and the stability of the Medigap market.^8^

Future research could consider the nuanced issue that Medigap is commonly offered by the same insurers that offer MA plans. If MA is more profitable for these insurers, they have less of a financial incentive to encourage beneficiaries to return to traditional Medicare. Descriptive work could assess whether private insurers offer both Medigap and MA plans, and the prevalence of each type of plan across the Medigap state-level types shows differences in benefits. For example, in guaranteed issue states, an insurer could choose to offer MA plans only or Medigap plans only to capitalize in 1 market or the other in a state where MA disenrollees are guaranteed the option to purchase Medigap at a community rate.

## Conclusion

As enrollment in MA continues to grow, and with MA currently providing coverage for the majority of Medicare beneficiaries,^20^ it is increasingly important to address “Medicare Advantage Trap.” In this paper, we find evidence that those who receive a top 10 highest-spending per beneficiary physician-administered drug are more likely to disenroll from MA in guaranteed issue states compared with states with no Medigap protections. When examining individual drugs, we find a larger association between MA disenrollment and Medigap state-level type for higher spending per beneficiary drugs with greater beneficiary out-of-pocket responsibility. To ensure Medicare coverage choice for high-need beneficiaries, state- and federal-level policies should make it easier for beneficiaries to switch between Medicare coverage.

## Supplementary Material

qxae136_Supplementary_Data

## Data Availability

The data in this analysis were shared through a data use agreement with the Center for Medicare and Medicaid Services. Per the data use agreement, that data are are not permitted to be shared.
