# Correction to: Medigap-guaranteed issue associated with Medicare Advantage disenrollment for beneficiaries administered a part B drug

**DOI:** 10.1093/haschl/qxaf116

**Published:** 2025-08-14

This is a correction to: Angela Liu, David Pittman, Gerard Anderson, Jianhui Xu, Medigap-guaranteed issue associated with Medicare Advantage disenrollment for beneficiaries administered a part B drug, *Health Affairs Scholar*, Volume 2, Issue 11, November 2024, https://doi.org/10.1093/haschl/qxae136

In the originally published paper, the authors incorrectly classified “Alaska” as a Medigap community-rating state when it should have been “Arkansas”. This misclassification, which originated from a prior *Health Affairs* article (Meyers et al. 2019), has been emended.

The appendix has been revised also.

The underlying analysis has been emended using the corrected classification. Relevant figure and tables are recreated and text emended accordingly. The authors confirm that the numerical changes are minor and the overall results and conclusions remain unchanged.

These emendations have been made in the article.

