# Supplementary material for: Medigap-guaranteed issue associated with Medicare Advantage disenrollment for beneficiaries administered a part B drug
Source: Health Aff Sch. 2024 Oct 23;2(11):qxae136. doi: 10.1093/haschl/qxae136 (PMC11538962; doi:10.1093/haschl/qxae136)
Supplement: qxae136_Supplementary_Data [file qxae136_supplementary_data.zip › Corrected Appendix_4.14.25.docx]

**Appendix**

***Supplemental Methods***

*Regression Specification*

The primary specification of our linear probability regression model is included below. Models (1) and (2) are used to generate the results in Figure 1.

Model (1):

Coverage_i,s_ = 𝛽_0_ + 𝛽_1_* Medigap state-level policy_i,s_ + ε, for beneficiary, i, who was administered a drug in our sample

Model (2):

Coverage_i,s_ = 𝛽_0_ + 𝛽_1_* Medigap state-level policy_i,s_+ ε, for beneficiary, i, who was not administered a drug in our sample

The dependent variable is a binary indicator of whether the beneficiary, i, was enrolled in MA for all 12 months in 2019 but not in 2020 (i.e., MA disenrollment). The main independent variable is a categorical, ordinal variable, where states are grouped into one of 3 categories: no Medigap consumer protection policies (42 states plus the District of Columbia), community-rating policy only (Washington, Minnesota, Vermont, and Alaska), or guaranteed issue (Maine, New York, Massachusetts, and Connecticut) (See Appendix Exhibit 3 for the states in each category). States with no Medigap consumer protection policies served as the reference group.

In addition the primary specification, we ran the model adjusted for beneficiary and zip code-level covariates. Models (3) and (4) present our adjusted models. We present results of the adjusted model in Appendix Exhibit 5.

Model (3):

Coverage_i,s_ = 𝛽_0_ + 𝛽_1_* Medigap state-level policy_i,s_ + 𝛽_2_Age_i_ + 𝛽_3_Sex_i_ + 𝛽_4_Race_i_ + 𝛽_5_(Poverty status) + 𝛽_6_(High school graduation rate) + 𝛽_7_(Elixhauser score)_i_ + ε, for beneficiary, i, who was administered a drug in our sample

Model (4):

Coverage_i,s_ = 𝛽_0_ + 𝛽_1_* Medigap state-level policy_i,s_ + 𝛽_2_Age_i_ + 𝛽_3_Sex_i_ + 𝛽_4_Race_i_ + 𝛽_5_(Poverty status) + 𝛽_6_(High school graduation rate) + 𝛽_7_(Elixhauser score)_i_ + ε, for beneficiary, i, who was not administered a drug in our sample

*Minimum volume threshold calculation*

The minimum volume was determined using a back-of-the-envelope calculation which should ensure compliance with the Center for Medicare and Medicaid Service’s Data Use Agreement. The minimum sample size that can be reported is a cell size of 11. First, we scaled that up given that we are using a 20 sample (11/0.2) to 55, then scaled given that 1% of beneficiaries disenroll (55/0.01) to 5,500, then scaled given that we have 3 unique groups (5,500*3) to a minimum sample size of 16,500.

**Appendix Figure 1**


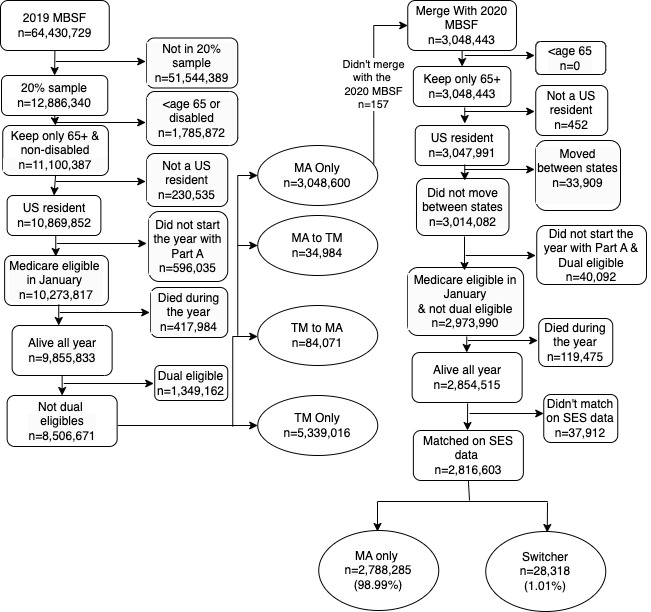


**Caption:** Flowchart of sample inclusion and exclusion criteria

**Source:** Medicare Beneficiary Summary File (2019-2020)

**Notes:**

**Appendix Table 1**

| **Brand Name**  **(Chemical Name)** | **Indication** | **HCPCS Code** | **Average**  **Spend Per Beneficiary** |
| --- | --- | --- | --- |
| **Opdivo**  **(Nivolumab)** | For adults with metastatic non-small cell lung cancer and tumors are positive for PD-L1, but do not have an abnormal EGFR or ALK gene. Used in combination with YERVOY (ipilimumab). | J9299 | $59,590 |
| **Keytruda**  **(Pembrolizumab)** | Immunotherapy treatment for several cancer types, including triple-negative breast cancer, colon or rectal cancer, melanoma, non-small cell lung cancer and others. | J9271 | $53,745 |
| **Herceptin**  **(Trastuzumab)** | For the treatment of early stage, HER2+ breast cancer; Patients are selected for therapy based on an FDA-approved test for Herceptin. | J9355 | $40,301 |
| **Orencia**  **(abatacept)** | Reduce signs and symptoms of moderate to severe rheumatoid arthritis in adults, polyarticular juvenile idiopathic arthritis and psoriatic arthritis in people ages 2 and older. | J0129 | $31,700 |
| **Alimta**  **(pemetrexed disodium)** | Indicated for locally advanced or metastatic nonsquamous non-small cell lung cancer and mesothelioma in combination with cisplatin. | J9305 | $25,404 |
| **Rituxan**  **(rituximab)** | In combination with methotrexate, adult patients with moderately to severely active rheumatoid arthritis who have had an inadequate response to one or more TNF antagonist therapies.    In combination with glucocorticoids, people ages 2 years and older with Granulomatosis with Polyangiitis, Wegener’s Granulomatosis, and Microscopic Polyangiitis.    Adults with moderate to severe pemphigus vulgaris. | J9312 | $24,758 |
| **Xolair**  **(omalizumab)** | Used to treat moderate to severe persistent asthma in people 6 years of age and older whose asthma symptoms are not well controlled, chronic rhinosinusitis with nasal polyps, food allergy in people 1 year of age and older, and chronic spontaneous urticaria in people 12 years of age and older. | J2357 | $22,345 |
| **Gammaked**  **(Immune Globulin Injection (Human) 10 Caprylate/Chromatography Purified)** | Indicated to treat primary humoral immunodeficiency in patients 2 years of age and older, idiopathic thrombocytopenic purpura in adults and children, and chronic inflammatory demyelinating polyneuropathy in adults. | J1561 | $21,238 |
| **Privigen**  **(Immune Globulin Intravenous (Human), 10 Liquid)** | Approved to treat types of primary immunodeficiency, raise platelet counts in patients over 15 with chronic immune thrombocytopenic purpura, treat chronic inflammatory demyelinating polyneuropathy in adults. | J1459 | $20,108 |
| **Infliximab** | Adults and children with multiple conditions, including Crohn's disease, ulcerative colitis, rheumatoid arthritis, psoriatic arthritis, ankylosing spondylitis, and plaque psoriasis. | J1745 | $18,878 |

**Caption:** Characteristics of the top 10 highest spending per beneficiary physician-administered drugs in 2019

**Source:** Medicare Part B spending dashboard

**Notes:** The average annual spending per beneficiary (column 4) is based on data for traditional Medicare beneficiaries, where prices and spending for physician-administered drugs are available. These spending per beneficiary numbers serve as a proxy for the spending per beneficiary that would be observed in MA, given that MA prices and spending on physician-administered drugs are not transparent.

**Appendix Figure 2**

**
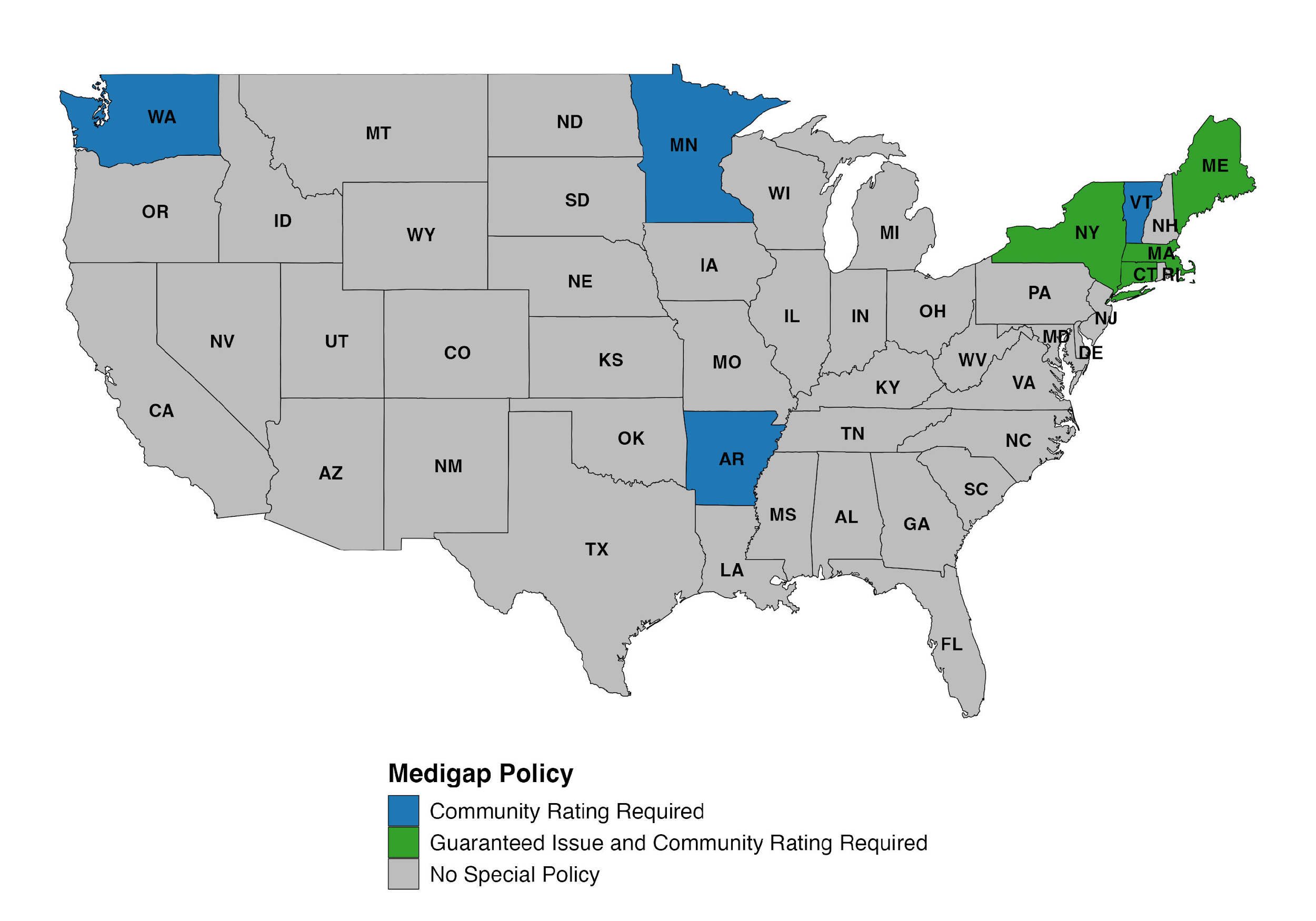
**

**Caption:** Categories of state-level Medigap policies in 2019

**Source:** Publicly available data on state-level policies

**Notes:**

**Appendix Table 2**

| **Drug** | **Number of Users** |
| --- | --- |
| Opdivo | 1,129 |
| Keytruda | 1,609 |
| Herceptin | 1,174 |
| Orencia | 805 |
| Alimta | 720 |
| Rituxan | 3,942 |
| Xolair | 752 |
| Gammaked | 631 |
| Privigen | 527 |
| Infliximab | 1,403 |

**Caption:** Number of beneficiaries by use of Part B drug

**Source:** Medicare Advantage carrier and outpatient encounter records (2019)

**Notes:**

**Appendix Table 3**

|  | **Administered a Top 10 Part B Drug in Our Sample** | | **Not Administered a Top 10 Part B Drug in Our Sample** | | **Not Administered a Top 150 Part B Drug** | |
| --- | --- | --- | --- | --- | --- | --- |
|  | **Coefficient** | **95% Confidence Interval**  **(p-value)** | **Coefficient** | **95% Confidence Interval**  **(p-value)** | **Coefficient** | **95% Confidence Interval**  **(p-value)** |
| **Total Number of Beneficiaries** | | | | | | |
| ***N*** | 12,064 | | 2,802,579 | | 2,768,801 | |
| **Disenrollment (ref: state with no Medigap protections** | | | | | | |
| ***Guaranteed Issue State*** | 3.73% | 2.62%-4.84%  (<0.001) | 0.332% | 0.13%-0.53%  (0.002) | 0.32% | 0.12%-0.52%  (0.003) |
| ***Community Rating State*** | 1.66% | -1.54%-4.87%  (0.302) | 0.08% | -0.49%-0.65%  (.773) | 0.08% | -0.48%-0.63%  (0.786) |
| **Age (ref: 60-65)** | | | | | | |
| ***71-75*** | 0.01% | -0.57%-0.61%  (0.957) | -0.35% | -0.41%-  -0.29%   (<0.001) | -0.35% | -0.41%--0.29%  (<0.001) |
| ***76-80*** | -0.62 | -1.11%-  -0.14%  (0.013) | -0.36% | -0.53%-  -0.20%   (<0.001) | -0.36% | -0.52%--0.20%  (<0.001) |
| ***81-85*** | -0.59 | -1.34%-  0.16%  (0.118) | -0.47% | -0.61%-  -0.33%   (<0.001) | -0.47% | -0.61%--0.33%  (<0.001) |
| ***>86*** | -0.45 | -1.29%-  0.40%  (0.291) | -0.41% | -0.56%-  -0.27%   (<0.001) | -0.40% | -0.55%--0.26%  (<0.001) |
| **Sex** | -0.041% | -0.60%-  0.58%  (0.974) | -0.04% | -0.09-  -0.01  (0.109) | -0.04% | -0.09%-0.001  (0.095) |
| **Race (ref: non-Hispanic White)** | | | | | | |
| ***Black*** | -0.03% | -0.79%-0.74%  (0.946) | 0.21% | 0.04-0.37  (0.014) | 0.21% | 0.05%-0.38%  (<0.05) |
| ***Hispanic*** | 0.50% | -0.72%-1.71%  (0.415) | 0.00% | -0.15-0.16  (0.984) | 0.01% | -0.15%-0.16%  (0.946 |
| ***Other*** | -0.09% | -1.11%-0.92%  (0.855) | 0.16% | 0.03-0.28  (0.016) | 0.16% | 0.03%-0.28%  (<0.05) |
| **Socioeconomic Factors (Zip Code Level)** | | | | | | |
| ***High School Graduation Rate*** | 1.58% | -0.73%-3.89%  (0.176) | 0.43% | -0.19%-1.05%  (0.169) | 0.48% | -0.08-1.05%  (0.091) |
| ***Below Federal Poverty Level*** | -0.72% | -6.48%-5.05%  (0.804) | 0.48% | -0.09%-1.05%  (0.095) | 0.44% | -0.18-1.05%  (0.161) |
| **Elixhauser Comorbidity Index** | 0.07% | -0.014%-0.16%  (0.098) | 0.003% | -0.01%-0.02%  (0.691) | 0.002% | -0.01-0.02%  (0.749) |

**Caption:** Appendix Table 5 shows the adjusted regression results, including coefficients on beneficiary and zip-code level covariates. The regressions are stratified by whether the beneficiary received a physician-administered drug in our sample or did not receive a physician-administered drug in our sample, Models (1) and (2), respectively. Model (3) shows a sensitivity analysis which includes beneficiaries who did not take one of the top 150 highest spending per beneficiary drug.

**Source:** The data were drawn from the 2019 Medicare Beneficiary Summary File (beneficiary-level covariates), 2019 and 2019 MA encounter and carrier data, and the 2016-2020 American Community Survey (zip-code level covariates)
